# Supercharged Smiles: A Discourse Analysis of Australian Media Coverage of Funding Dental Care Through Superannuation

**DOI:** 10.1111/adj.70002

**Published:** 2025-08-15

**Authors:** A. C. L. Holden

**Affiliations:** ^1^ Sydney Dental Hospital and Oral Health Services, Sydney Local Health District Surry Hills New South Wales Australia; ^2^ The University of Sydney School of Dentistry Faculty of Medicine and Health Surry Hills New South Wales Australia

**Keywords:** cosmetic dentistry, discourse analysis, ethics, oral health, superannuation

## Abstract

**Background:**

The early use of superannuation savings towards dental treatment is a phenomenon that has emerged in recent years within Australia. This research investigates the media's response and coverage of how this usage of private, yet segregated funds has developed and grown. Through analysing the media's portrayal of this issue, valuable insights into dentists' evolving professionalism and societal attitudes towards oral health can be gained.

**Methods:**

A search of two online databases and the grey literature was used to identify relevant media sources that related to the utilisation of superannuation funding to facilitate accessing dental care in the Australian private sector. A discourse analysis methodology was used to analyse the corpus of articles identified through the search strategy.

**Results:**

A total of 36 separate, non‐duplicate media articles were located through the search strategy. The discourse within the texts was examined and the key themes and power dynamics that emerged were explored in detail. The three themes used to structure the discussion of the corpus of articles were: (1) outrage at the necessity of compassionate release of superannuation for essential dental care; (2) the abuse of the compassionate release of superannuation for elective and cosmetic dental care; and (3) exploitation of vulnerable consumers by professionals.

**Conclusion:**

The overarching discourse relating to early superannuation access to fund dental care was negative, with media articles covering instances of consumers losing their money, having to pay more tax as a result of accessing their superannuation early than they were expecting, and the reality that accessing superannuation early has significant financial impacts later in life. Dentists and third‐party access facilitators were portrayed as being exploitative and lacking in transparency in relation to the costs involved in early superannuation access. Dentists were also suggested to be aiding in the inappropriate access of superannuation funds for treatment that was largely elective and cosmetic in nature, rather than being more essential in nature in alignment with the intentions of the early compassionate release scheme.

**Abbreviations**
ADA: Australian Dental Association
ATO: Australian Taxation Office
CDBS: Child Dental Benefit Schedule
CDDS: Chronic Diseases Dental Scheme
CRS: compassionate release of super

**Summary**

- This research serves to illustrate the ethical, social and professional challenges that the early release of superannuation for dental treatment presents to dentists.
- Those who encounter patients who plan to access their superannuation or wish to facilitate consumers utilising their retirement savings to fund their private dental treatment need to be able to communicate the risks to patients effectively.
- While patients may argue that their superannuation savings are theirs to use as they wish, dentists have a wider obligation to society to consider whether proposed treatment truly fits within the intention of the early compassionate release scheme.
- This research also highlights significant concerns in relation to how accessing superannuation early might be advertised in the context of funding dental care.

## 1 Background

With only a small proportion of the Australian population eligible and able to access publicly funded dental care, the public mainly rely on the private dental sector when seeking dental treatment. The barrier of affordability to accessing dental care is well documented and understood as a significant reason for the avoidance of dental treatment within Australia, with 32% of adults reporting avoiding dental care due to cost [1]. Recent publicly funded initiatives have been shown to increase access, with good engagement from the dental profession to participate, with the Chronic Diseases Dental Scheme (CDDS) being a prime example where both public and profession utilised the scheme extensively [2, 3, 4]. The most recent publicly funded scheme, the Child Dental Benefit Schedule (CDBS), has been less well embraced by the public, being restricted in the scope of care provided on the schedule and being limited to those under 18 years of age [5, 6].

**FIGURE 1:**
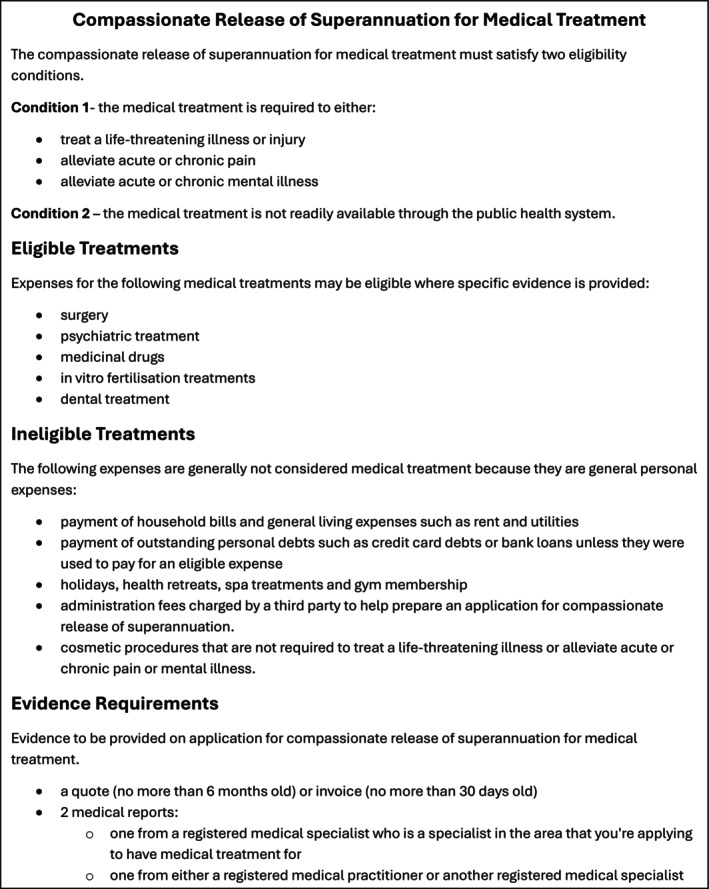
Compassionate release of superannuation for medical treatment.

Since 1992, the Superannuation Guarantee has mandated that a percentage of workers' earnings be offset from wages and contributed to a segregated fund, inaccessible until retirement. The Australian Taxation Office (ATO) allows individuals to access their superannuation early for compassionate reasons, if certain criteria are met, including funding urgent healthcare. The release of superannuation for medical expenses is set at a high threshold; the eligibility criteria for compassionate release of super (CRS) for medical reasons are outlined in Figure 1. Dentists may support patient requests to the ATO for CRS for qualifying dental treatment. There have been increasing instances of those seeking dental care using their superannuation funds to access dental treatment, with the numbers of applications for CRS growing from 7140 in 2018–2019 to 31,780 in 2023–2024. Those applications that are approved by the ATO (just over 70% of the total applications in 2023–2024) represent $526.4 million in funds released early for dental treatment [7]. This process has proved to be controversial, with reports in mainstream media of abuse, along with concerns that members of the public are not fully aware of the financial implications of accessing their superannuation early.

The professional implications of health professionals being involved in early superannuation access can be felt no more clearly than through the National Boards of both Medicine and Dentistry, the Medical Board of Australia and the Dental Board of Australia, releasing a joint statement on professionals who are regulated by these boards being involved in early superannuation access. In their joint statement, the Boards state; ‘The significant increase in approvals for the use of CRS for dental treatments in recent years, raises concerns that some practitioners may be placing profits over patient care’ [8]. A year before this joint statement, the Dental Board of Australia had separately published a statement urging compliance with advertising rules in relation to accessing superannuation early [9]. Despite these concerns from regulators, the peak representative body for dentists in Australia, the Australian Dental Association, has publicly asserted its belief that dentists are behaving responsibly and complying with AHPRA's advertising rules [10].

This research reviews media reports that relate to superannuation access and dental care. The purpose of this analysis is to explore how this privately funded process to facilitate access to necessary care is being discussed and impacting the professional practise of dentistry, including the public perception of dental professionals. Of particular interest in this research is how the discussion of the issue of early access to superannuation portrays the relationship between the dental profession and health consumers in respect of power dynamics and professionalism. The insights gained from this research will help to inform policy approaches to how superannuation might be accessible for oral health reasons, and to assist in synthesising the ethical and professional issues presented by dentists participating in facilitating private individuals accessing their superannuation funds early. Because of the nature of the insights being sought through this analysis, a discourse analysis will be carried out to reveal the innate and underlying discourses within the texts identified [11]. By applying a methodological approach that is focused upon the social and relational aspects of dentistry and the early, compassionate release of superannuation, the insights will be particularly well oriented to inform the future direction of professional and regulatory policy.

## 2 Methods

This research employed a qualitative approach to analysing the media surrounding dentistry and early superannuation access. Media articles were accessed and identified through identical searches using two separate databases: (1) ProQuest Central and (2) Factiva. The search was then replicated within Google News as a grey literature search. The search terms used were the Boolean terms: (dental OR dentistry) AND (super OR superannuation). This search strategy returned 1602 initial results across the three searches which were then reviewed for relevance.

As this research pertains to the specific phenomenon of Australian consumer and professional behaviours around superannuation early access and usage, articles were included if they referred to early superannuation access and dental care in Australia. The search allowed for the potential that international media outlets may have covered this issue in Australia with the search being unrestricted to location of article origin. Articles were excluded if they did not relate to superannuation being used to fund dental care, did not relate to Australian superannuation or if the full text of the article was not available. A wide range of media were included; online newspaper articles, radio interviews (transcripts), online blogs and articles by independent organisations were incorporated into this analysis. There were no date parameters applied to this search, noting that given this is a contemporary issue, the results of the search were likely to be bound within a narrow date range.

Media articles were subjected to discourse analysis, reviewing the texts to reveal relational themes and contrasting narratives relating to how superannuation is being used to fund dental treatment was presented and discussed within media articles. Discourse analysis is a qualitative approach that employs critical reading and re‐reading of the constituent texts to develop insight into power relationships, as well as the social, political, and cultural contexts of this particular professional issue within dentistry [11, 12].

This application of discourse analysis follows the approach taken by Lupton in one of the first uses of this methodology to analyse data in a health context [13]. More recently, discourse analysis has been used in media analysis relating to oral health exposure in news stories [14]. This application of the methodology provided qualitative insight into how the dental profession and oral health are depicted in news stories, demonstrating insight into the perceptions of the public and society into dentistry and the value of oral healthcare. The value of using discourse analysis in this research is that the consideration of how the wider sociological, political, and contexts of the texts are captured within the qualitative evaluation [12]. Within this, power relations are of key importance; how these are spoken about and portrayed within the texts will be examined within this exploration.

The objective of this research is to examine the issue of early access, compassionate release of superannuation to fund dental treatment from the perspective of its impact on public health and the professional position of dentistry and those who provide oral healthcare [15]. Lupton identified discourse analysis as having particular value in conducting research into power relationships within texts. In this evaluation, this will be applied to examine the power relations that exist between the stakeholders in the media articles, in particular those between health professionals and health consumers [13].

Discourse analysis has been criticised for being methodologically vague [12] and as an emergent approach in qualitative research [16]. It is important to acknowledge that this analysis does not claim any universal truth, with the research approach being one of analytical modesty, with the researcher's own experiences and bias making an undeniable contribution to the analysis [16]. As per Tonkiss' commentary upon the objectives of discourse analysis, this exploration of the media items relating to dentistry and early release of superannuation funds seeks to be persuasive rather than making any claims to being a single truth [11].

Institutional ethics approval was not applied for in this research, as this analysis utilises publicly available media and texts, with no participant data being collected or used.

## 3 Findings

**FIGURE 2:**
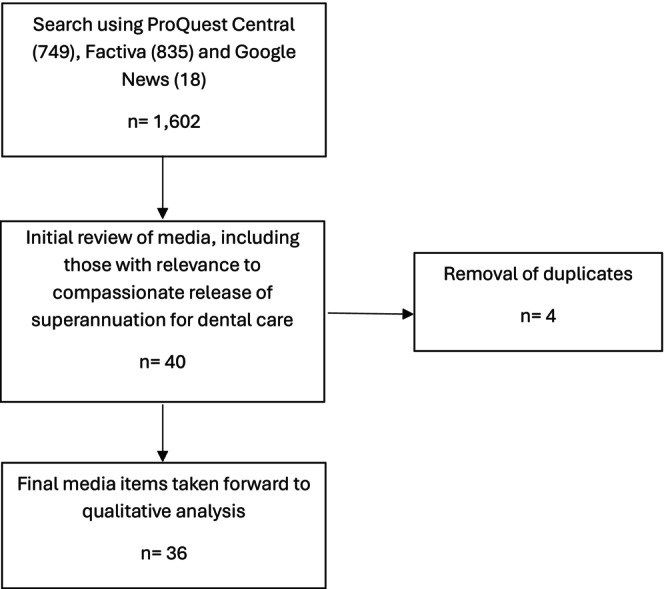
Search strategy used to identify media items for analysis.

The media search was completed in June 2025. Utilising the search strategy described above, and following the removal of duplicate articles (*n* = 4), where publishing outlets had reprinted the same article under a different publication title (e.g., The Sydney Morning Herald and The Age), a total of 36 separate media items relating to superannuation being used to fund dental care were identified. The search strategy employed in this research is illustrated in Figure 2. Some articles covered similar issues and stories, whilst not being true duplicates. These similar stories were not excluded from the analysis, but close relationships between sources within the corpus are reported throughout the findings. The corpus comprises 36 separate, often related articles, written by 25 different authors, and published across 18 media platforms. The articles range from 2022 to June 2025.

Within the corpus, there are several repeated aspects to the discourse around the CRS for dentistry. These are: (1) outrage at the necessity of CRS for essential dental care; (2) the abuse of the CRS for elective and cosmetic dental care; and (3) exploitation of vulnerable consumers by professionals. These interrelated components of the discourse serve to provide structure to the presentation of the findings.

### 3.1 Outrage at the Necessity of CRS for Essential Dental Care

The need for consumers to seek CRS for dental care is discussed within the corpus as being a symptom of a health system that neglects oral health, with consumers being forced to access alternative ways of funding expensive yet necessary dental care. Some of the reasons for dental care being particularly prevalent as a reason for early CRS are due to delayed care because of restricted access during the COVID‐19 pandemic, leading to more complex and expensive dental care needs [17, 18]. Some of the articles, especially the oldest within the corpus, report on the belief from health policy experts that the CRS for dental care masks the wider issue of poor provisions for oral health within the existing provisions in the health sector:

It is outrageous that Australians are taking $1.6 billion dollars out of their super funds to pay for care that should be available relatively quickly in the public sector [17].

The Self‐Managed Super Fund Association shared its view that the use of CRS for essential health treatments was symptomatic of the health system having gaps that needed addressing, rather than superannuation being used as a substitute for universal health coverage:

In the face of [the number of CRS applications], perhaps the primary should be on reforming Medicare and the funding of our healthcare system to better support the provision of necessary treatment rather than shifting the financial burden onto individuals' retirement savings [19].

One of the repeating themes within the corpus is the vulnerability of those who draw from their superannuation savings to access treatment, with the reality of consumers' situations being well‐illustrated by this same article:

So if you're living in pain, then you've got to make a decision about, “Well, I don't have the savings. Where can I get the money from?” Do you draw down on your mortgage? Or do you take money out of your superannuation? That's really the only option that's available to you [17].

It is this vulnerability that heightens the negative portrayal of those in the corpus who are positioned as profiting from this demographic of consumer. In another article, a dentist reported on her experiences in managing patients who had delayed care during the pandemic:

We saw a lot of delayed care, and as a result, the oral health of a lot of our patients had deteriorated…with toothache, a lot of the patients that we had seen, the gum disease had progressed a lot faster [18].

This article also noted the stark vulnerability of consumers accessing their retirement funds for the purpose of seeking care, especially care that might not strictly fit within the intent and purpose of the CRS process:

People seeking medical care are vulnerable and can be affected by physical or emotional pain. They are being taken advantage of and have little recourse if the procedure fails or that are complications. If it goes wrong, your money is gone, and you have lost all your retirement savings [18].

One article both criticised the lack of support for essential dental work from Medicare, and the unplanned expenses resulting from an early CRS withdrawal to have care provided that was caused by childhood trauma. Stories that related to the early CRS and the expense involved in this attracted comment and ire from politicians within the articles of the corpus, in this example, past Greens leader Adam Bandt used the example of a consumer spending $67,000 of his superannuation as an example of why dental care needed to be included within Medicare [20]. The reality of a $67,000 withdrawal, including additional taxation expenses, is that the total impact was a $80,000 loss of saving which compounds to $300,000 over the course of that person's working life, leaving a lot less in retirement [20].

The Australian Dental Association is represented as supporting the expansion of publicly funded oral healthcare, with frustration being voiced in an interview that further investment had not been made, resulting in the reliance of consumers on avenues such as CRS to access dental care:

I'm extremely disappointed. We've tried to say to government, OK, let's target funding at those most at need, those most disadvantaged. Let's do it now because we know there's a real problem out there. A Royal Commission and Senate select inquiry have found those findings. It's been three years we've been thumping the table over this saying, come on [21].

Within the corpus, the long‐term concern that individuals will be unable to retire due to having too little saved is frequently encountered. The impact of this on society as a whole is recognised, with this practice of having prematurely accessed superannuation savings meaning greater levels of retirement and pension support will be needed for those reaching old age in the future [22].

### 3.2 The Abuse of the CRS for Elective and Cosmetic Dental Care

Articles within the corpus were sceptical of whether CRS for dental care was legitimate in the context of the conditions of the scheme to release funds early. The then‐President of the Australian Dental Association is reported to take a clear stance on the limitations to accessing superannuation for dental care:

You need acute or chronic pain, or significant functional disability, you know, can't eat properly…that doesn't mean you get veneers on your front teeth, you straighten your teeth, you get fancy dental treatment; you just get basic care, really through this scheme [23].

This attitude is supported by several others within the corpus, with one financial expert theorising that large CRS withdrawals are signifiers that care didn't fit within the intentions of the scheme:

AMP chief economist Shane Oliver said when people were spending such a significant amount of money for dental, the work was likely for cosmetic procedures, not essential work [24].

The media also identified that there were plentiful examples of dental advertising that contrasted with the ATO's intentions, with clinical advertising early CRS as a way for consumers to achieve their ‘Dream Smile’ [24], with this being a far cry from the life‐threatening medical conditions, chronic pain or chronic mental illness justifications given by the CRS scheme. This article provides the text from one particular Facebook advert:

Tired of waiting for your dream smile? Access your super early and wait no more! [24]

Similarly, the Self‐Managed Super Fund Association stated in an article that the association did not believe that many of the applications for CRS met the intention and spirit of the scheme, which was underpinned by the principle of last resort, not using the scheme to access elective, cosmetic treatment [19]. The reporting of the demise of the NSW dental clinic chain, Supercare Dental and Cosmetics, notes that the business advertised in a way that was misaligned with the intention of the ATO:

[M]ultiple Supercare Dental and Cosmetics advertisements spruiked that dental implants and porcelain veneers “to achieve the smile you've always wanted” were “potentially affordable through your super fund” [25].

Reporting on the joint statement from the Medical and Dental Board of Australia on CRS for medical and dental treatments, Bite Magazine (a dental industry trade publication) reported that the Medical and Dental Boards of Australia would be working closely with the ATO to identify inappropriate practice:

Under close examination is the practice of practitioner with high rates of report writing that indicate inappropriate patient assessment may be occurring [26].

One article described the use of superannuation savings as a ‘moral hazard’ [27] where the ATO scheme for early CRS, designed to support exceptional instances involving chronic illness, was being used to fund discretionary wants, no less from young and vulnerable households. The article notes that currently just under 10% of restorative and specialist dental revenue comes from consumers' super balances, with an expectation that this will rise to 25% by 2027. While the article is heavily focused towards discussing the opportunistic behaviour of third‐party companies that charge consumers to facilitate their superannuation fund access, the title is directed at dentists' role in what is portrayed as a dishonest practice, ‘Start‐ups, dentists drain retirement savings in “super scam”’.

Within the corpus, consumers are generally portrayed as victims in this process, as naïve and financially inexperienced patients who are misled into taking action that is sold as accessing ‘free money’, but which has the potential to be financially ruinous in the future. While it is the third‐party facilitators and dentists who are described as ‘greedy’ and ‘shonky’ [22], consumers are also described as conducting ‘raids’ [17, 20, 28, 29, 30, 31] on their superannuation in some articles, referring to the potentially illegitimate or even illegal access for non‐justified reasons.

The conflated nature of oral health, whereby cosmetic/aesthetic considerations cannot be separated from other health aspects of dental treatment, is raised by some (notably cosmetic dentistry providers) as part of the justification being relied on as to why seemingly cosmetic procedures are being approved from CRS:

Dental Boutique said a range of medical treatments could involve a cosmetic element, adding porcelain restorations, such as crowns and bridges that improved dental aesthetics were part of a holistic treatment for conditions like TMJ disorder [32].

Regardless of where one considers the boundaries between essential dentistry and cosmetic treatment to lie, the narratives within the corpus do not speak of cosmetic enhancement as a welcome by‐product of treating chronic dental pain, but as the driving force behind treatment being sought initially.

### 3.3 Exploitation of Vulnerable Consumers by Professionals

There was strong discourse within the corpus in relation to the belief that health professionals were exploiting their patients in relation to CRS:

Financial Services Minister Stephen Jones this week said some surgeons and medical practitioners consider their patients' super as a “personal river of gold” and accused them of “unconscionable behaviour” by encouraging them to access retirement income for medical treatment. “They are encouraging, and even pressuring, patients to tap into their super for what might be termed life enhancing procedures like cosmetic surgery. There are business models set up to game the system. This is deeply troubling, and I am calling this out,” [18]

The articles from trade publications that reported on the joint statement on early CRS made by the Medical and Dental Boards of Australia demonstrated the national regulators' concern relating to the practise of early superannuation withdrawal. The narrative of the Agency's CEO against practitioners who are taking advantage of consumers is forceful and unambiguous:

We are deeply concerned by reports that some practitioners may be putting their own financial gain ahead of their patients' best interests, Ahpra CEO Justin Untersteiner said. ‘We're working with the ATO any potential predatory practice. Practitioners are on notice that we will take action to protect the public.’ [26, 33]

The tacit implication of this statement from the regulator is that there is a corresponding rise in complaints from consumers relating to care received that has been funded by early superannuation withdrawals. Along with having financial vulnerability, many of the consumers are also portrayed as being socially vulnerable also, illustrated through their histories and how these are presented within the corpus:

Tully Denahy destroyed his teeth through years of hard living – and fixed them at age 36 by draining his superannuation account dry…He says his teeth were damaged through a lack of care after he lived on the streets as a teenager, existed on sweets, used drugs, and did not own a toothbrush until he was 26 [23].

The corpus presents consumers as being vulnerable to exploitation by dentists, but also third‐party agents who assist those seeking to make CRS applications, charging a fee to act as facilitators to the process. The corpus presents these third parties as misleading their customers as to the consequences of early CRS:

Will this affect me later in life?

Negative! There are no penalties of fees for using your Super early, as long as you go through approved health providers with a treatment plan [23].

Commentary within the corpus attributes the rise in CRS applications to be both a sign of escalating costs of living, and that third‐party agents in the process are not taking the need for financial consent seriously:

I think what it demonstrates is there's a real cost of living crisis out there but also what we've found in this investigation is that there are third parties out there coaching people to take their money out early and not informing them of the repercussions and potentially encouraging them to take it out in situations where they wouldn't otherwise be allowed to access that money [34].

This repeated reference to consumers being vulnerable and at risk of exploitation demonstrates the perception that dentists (and other health professionals), as well as predatory commercially‐driven companies are abusing their positions in encouraging already vulnerable people to place themselves into greater positions of vulnerability. Within one article, a cosmetic dentist interviewed from the chain, Dental Boutique, attempts to separate the clinical provision of cosmetic services by his clinic from the process of engaging a third‐party facilitator to help patients to access their superannuation:

Once we've done our jobs as medical professionals, then it's up to the third party agents to decide whether or not the patient is applicable for super [23].

The sincerity of a dentist distancing themselves from the CRS process is this way is questionable, especially when another article covering multiple instances of patients from Dental Boutique who were complaining about the clinic's practises, references some of Dental Boutique's social media advertising which clearly promoted superannuation is an option for pursuing cosmetic care:

It could be Invisalign that you're looking for, it could be veneers that you're looking for…we will do whatever it takes to help you by looking at payment options, payment plan, and superannuation as well. Don't worry about the major cost [32].

The negative impact on individuals' future financial well‐being, and the lack of perceived clarity from both dentists and third‐party facilitators on CRS's impact on future prosperity was heavily criticised throughout the corpus:

Financial adviser Dawn Thomas said she was “baffled” by the online testimonials on web pages that don't disclose key information about financial risks [28].

While different articles in the corpus give different examples of how early CRS impacts financial wellbeing, one of the most compelling examples is given as $38 billion in early withdrawals under the scheme will lead to $85 billion of losses from final superannuation balances [28]. The underlying suggestion within the corpus being that irresponsible CRS is not just personally irresponsible but also represents a liability to society.

Consumers are reported within the corpus as being surprised and unaware of the cost of an early CRS application being approved, both in the context of the resultant financial outcomes later in life, but also the immediate income tax implications meaning that the amount taken from their superannuation balance is far greater than the amount requested:

No‐one made me aware of that [in advance]. I just didn't even think. It's my money [20].

Within the corpus, a series of articles from 2025 covered the story of two dentists in Western Australia who were suspended pending legal action, leaving multiple patients in a position of limbo with their treatment. The articles focused on the vulnerability of the patients who were left having paid money for treatment that hadn't been provided and dentists who were unable to assist them. As the stories develop, it becomes apparent that CRS withdrawals facilitated the care in many instances, with the dentists themselves being reported to have encouraged patients to take this avenue:

Ms Smoje said Dr Mahadeva recommended getting both upper and lower teeth done, but she could not afford the $45,000 quoted price so he suggested accessing her superannuation and discounted his price to $37,000. By March 2023 her application was successful and she had paid Dr Mahadeva a total of $28,000, although she said she was not warned an extra $8,000 would also come out of her super in tax [35].

Within the series of articles covering these patients' experiences, there are numerous similar instances where patients had suffered the same challenge of having paid their money and not received the treatment that they had expected. The financial loss and impact on these consumers is clear:

I lost all my benefits I planned for my kids…I feel quite disgusted. That was my super [36].

One of the dentists involved, Dr. Mahadeva, spoke to the journalists covering this story and his words do show insight into his former patients' plight:

These individuals are trapped in a vulnerable position – clinically, emotionally, and financially – aggravated by them living in a remote region [36].

His position is that he is similarly a victim in this scenario, a victim of Ahpra's over‐regulation who had refused to grant him conditional registration to allow him to finish these patients' treatment. One of the aspects of this story arc that is not well highlighted within the article series is the fate of one of the titular patients, Ms. Smoje:

Since sharing her story with the ABC, Ms Smoje has been offered help from a clinic in Kalgoorlie who told her they would complete her implants for free. “It's amazing. My faith's been restored in humanity.” she said [36].

Another series of articles covered the collapse of Supercare Dental and Cosmetics, a group dental practice chain that left patients who had paid for their treatment without care. While many patients were impacted, the story focuses on the individual circumstances of Ms. Amourous, described in a way as to emphasise her potential vulnerability:

The cost [of treatment] was $48,000, but they reassured her they would help the single mother of four apply to the tax office to access her superannuation [25].

The company, Supercare Dental and Cosmetics, is described as a predatory and aggressive business that traded whilst insolvent, pushed patients towards early accessing their superannuation and who exploited both patients and staff who had worked for the clinics. The patients are noted to be mainly lower‐to middle‐class with little financial literacy or ability to be able to access funding for care other than their superannuation.

## 4 Discussion

The three converging narratives within the corpus coalesce to form an overarching discourse around the neglect of oral health in Australia's health system and supports previous commentary that considers this aspect of dentistry to be a free market that has arisen to service social trends for an ‘ideal’ aesthetic standard of straight, white teeth [37, 38, 39, 40, 41]. Within the nexus that has emerged, we find dentists who have commercialised the socially attractive ideal dental standard and who behave in a manner that is less professional and more transaction focused. Allied with dentists are those purely business‐focused entities who exchange facilitating superannuation access for a set fee. The corpus is a narrative of loss and damage; patients and consumers who have lost the chance of future opportunity for the immediate gratification of aesthetics and dentists who have lost their standing within society with their professionalism questioned.

The conflation of cosmetic and essential dental treatment is common within the corpus, both overtly as part of the question of what type of dental treatment truly meets the ATO eligibility criteria for CRS, but also tacitly through the absence of basic and routine care. The ADA is reported as giving clear comment as to what care they feel meets this standard, as the body representing the dental profession within this discussion [23]. In the media stories analysed, not a single case of basic dentistry is reported, with even those that are reported as legitimate cases for CRS being complex implant cases. In the discussion that early CRS represents an underlying absence of Medicare support for dentistry, the assumption within the corpus is that dentistry's inclusion within the wider Medicare scheme would negate the need for patients to access their care through utilisation of superannuation in the way that is presented in the media explored. Recent research has investigated how an expanded Medicare might be designed and be implemented, including what types of care might be covered [42]. The complex scope of care reported within the corpus—implants, orthodontics, and cosmetic veneers—are not likely the first treatments to be included in a future dental scheme. What the ATO has created is a privately and individually funded scheme that helps to drive complex and cosmetically focused dental care. There is no breakdown on how the ATO applications might be structured in terms of the types of treatment funding is being sought for (i.e., essential management of chronic pain and loss of function or cosmetic care), only that the eligibility criteria appear to be either loosely applied or policed. This privately funded scheme will have financial implications for wider society in the longer term, either though consumers being financially unstable in later life and requiring more public support, or through becoming eligible to seek care and complex remediation through the public oral health services due to financial hardship.

The discourses within the research portray a powerful dental profession that holds trust from society, and that the public wants to engage services from. Members of the professional community are damaging that relationship through the embracing of free‐market and commercialised behaviours that involve promoting cosmetic and treatment aspirations through misleading advertising, with a lack of true financial consent underpinning the care delivered. The corpus portrays the dental profession as being either directly or complicitly abusive of their position of power and responsibility in how they are interacting with consumers in the context of superannuation. Health consumers are often automatically assumed to be disempowered due to the nature of seeking care and relying on professional expertise and knowledge. However, in this instance, patients are in a position of financial disadvantage and are in a position of even greater disadvantage.

This analysis contributes to the growing body of work around how the dental profession is acting as a commercial determinant of health [43], with the selling of care helping dentists to fill their time within a climate of financial uncertainty [44]. The corpus deals with the immediate gratification of cosmetic care and the long‐term negative financial implications of this in retirement. What is missing from the discourse is discussion that for many of the cases featured of young consumers embarking on complex care, the dental care they receive through their superannuation funds will likely need expensive and extensive maintenance and replacement over the course of the rest of their lives. This absence amplifies the mystery of dental care that is already presented in the discourse, with the lack of transparency around the financial impact of CRS being further accentuated.

## 5 Conclusions

Through discourse analysis, this research provides greater insights into the social phenomenon that is the compassionate release of superannuation to enable dental treatment. Through this analysis, it is possible to conclude that while in principle the utilisation of superannuation funding for dental care is an exercise in consumer autonomy, the surrounding considerations find this phenomenon to be highly problematic. Firstly, there is the clear lack of information and financial consent for consumers accessing their superannuation money in this way, both in the immediate impact on income tax and also in relation to future financial and social well‐being. Part of the purpose of superannuation is to ensure the future well‐being of citizens, but also to ensure that an ageing and growing population is financially sustainable and does not place too much of a burden upon the Australian welfare system. If consumers are able to use a scheme intended to offer a safety net for significant financial distress posed by serious medical issues for elective care, this presents an uneasy interaction with the intention of the scheme to alleviate the financial burden on old age upon the state and broader society.

Another finding of this analysis is the almost‐entirely negative way that dentists are reported to be exploiting both the superannuation system and patients in promoting and facilitating early CRS for elective dental treatments. The dental profession often laments its portrayal in the media, and this analysis provides another example where the profession is damaged by its close association to commercial motivations and business models, where dentists appear numb to the negative impact to patients' long‐term holistic wellbeing. While the articles focus on a small number of extreme cases, the fact that withdrawals for dental have so markedly grown, adding hundreds of millions of dollars to the amount spent on dental care; it would not be correct to argue that this is an isolated or insignificant challenge for dentistry's professionalism.

## Conflicts of Interest

The author declares no conflicts of interest.

## Data Availability

The data that support the findings of this study are available from the corresponding author upon reasonable request.
